# Low miR-150-5p and miR-320b Expression Predicts Reduced Survival of COPD Patients

**DOI:** 10.3390/cells8101162

**Published:** 2019-09-27

**Authors:** Andreas Keller, Nicole Ludwig, Tobias Fehlmann, Mustafa Kahraman, Christina Backes, Fabian Kern, Claus F. Vogelmeier, Caroline Diener, Ulrike Fischer, Frank Biertz, Christian Herr, Rudolf A. Jörres, Hans-Peter Lenhof, Robert Bals, Eckart Meese

**Affiliations:** 1 Chair for Clinical Bioinformatics, Saarland University, 66123 Saarbrücken, Germany; tobias.fehlmann@ccb.uni-saarland.de (T.F.); muka.uni@gmail.com (M.K.); c.backes@mx.uni-saarland.de (C.B.); fabian.kern@ccb.uni-saarland.de (F.K.); 2 School of Medicine Office, Stanford University, Stanford, CA 94305, USA; 3 Department of Human Genetics, Saarland University Hospital, 66421 Homburg, Germany; n.ludwig@mx.uni-saarland.de (N.L.); Caroline.Diener@uni-saarland.de (C.D.); ulrike.fischer@uniklinikum-saarland.de (U.F.); Eckart.Meese@uks.eu (E.M.); 4 Center for Human and Molecular Biology, Saarland University, 66421 Homburg, Germany; 5 Department of Medicine, Pulmonary and Critical Care Medicine, University Medical Center Giessen and Marburg, Philipps-University Marburg, Member of the German Center for Lung Research (DZL), Baldingerstrasse, 35043 Marburg, Germany; claus.vogelmeier@med.uni-marburg.de; 6 Institute for Biostatistics, Hannover Medical School, 30625 Hanover, Germany; Biertz.Frank@mh-hannover.de; 7 Department of Internal Medicine V–Pulmonology, Allergology, Intensive Care Medicine, Saarland University Hospital, 66421 Homburg, Germany; Christian.Herr@uks.eu (C.H.); robert.bals@uks.eu (R.B.); 8 Institute and Outpatient Clinic for Occupational, Social and Environmental Medicine, University Hospital, LMU Munich, Comprehensive Pneumology Center Munich (CPC-M), Member of the German Center for Lung Research (DZL), Ziemssenstrasse 1, 80336 Munich, Germany; rudolf.joerres@med.uni-muenchen.de; 9 Center for Bioinformatics, Saarland University, 66123 Saarbrücken, Germany; lenhof@bioinf.uni-sb.de

**Keywords:** miRNA, COPD, cancer, survival

## Abstract

Chronic obstructive pulmonary disease (COPD) is associated with an increased risk of death, reducing life expectancy on average between 5 and 7 years. The survival time after diagnosis, however, varies considerably as a result of the heterogeneity of COPD. Therefore, markers that predict individual survival of COPD patients are of great value. We analyzed baseline molecular profiles and collected 54 months of follow-up data of the cohort study “COPD and SYstemic consequences-COmorbidities NETwork” (COSYCONET). Genome-wide microRNA signatures from whole blood collected at time of the inclusion in the study were generated for 533 COPD patients including patients that deceased during the 54-month follow-up period (*n* = 53) and patients that survived this period (*n* = 480). We identified two blood-born microRNAs (miR-150-5p and miR-320b) that were highly predictive for survival of COPD patients. The expression change was then confirmed by RT-qPCR in 245 individuals. Ninety percent of patients with highest expression of miR-150-5p survived the 54-month period in contrast to only 50% of patients with lowest expression intensity. Moreover, the abundance of the oncogenic miR-150-5p in blood of COPD patients was predictive for the development of cancer. Thus, molecular profiles measured at the time of a COPD diagnosis have a high predictive power for the survival of patients.

## 1. Introduction

Chronic obstructive pulmonary disease (COPD) is often associated with comorbidities [1] and an increased risk of cancer [2,3]. It affects several hundred million people worldwide and represents one of the most common causes of death. The underlying pathophysiological mechanisms are highly complex and involve a multitude of diverse factors including chronic inflammation, increased levels of oxidative stress resulting in DNA damage, cytokines repressing DNA repair mechanisms [3], and changes of the pulmonary microbiota [4]. Causative factors include environmental stress with the consumption of tobacco as the main cause of COPD [5]. There are also multiple genetic factors that have been associated with COPD, in particular single nucleotide variants identified by GWAS studies [6,7,8]. Mounting evidence indicates that single nucleotide polymorphisms in RNAs that are not translated into proteins also play a role in the development of COPD [1,9,10]. In particular, small non-coding RNAs (sncRNAs), such as microRNAs (miRNAs), that are 21−23 nucleotides in length, apparently have an impact on the development and progression of COPD. Tissue-based miRNA signatures as well as circulating miRNA profiles in COPD have previously been reported [11,12].

Blood-born miRNAs offer themselves as potential minimally invasive biomarkers in COPD patients. While many studies on miRNA biomarker for lung diseases rely on case-control setups [13], we recently evaluated miRNA profiles of COPD patients towards their potential to predict lung cancer development in a longitudinal setup [14]. Utilizing the German COPD and SYstemic consequences-COmorbidities NETwork (COSYCONET) study that has collected biomaterial and clinical data of COPD patients for a follow-up period of currently 54 months, we identified blood-born miRNAs that showed altered abundances in COPD patients who developed cancer [15].

Compared to the increase of knowledge about clinical and molecular factors associated with the development of COPD in general, less knowledge has been gathered on factors underlying the marked variance in survival after a diagnosis of COPD. Remarkably, the reduced survival of COPD patients appears to be independent of coexisting cardiovascular and metabolic disorders [16]. Only a few factors, including airway obstruction indicated by low forced expiratory volume in 1 s (FEV_1_) and gas uptake indicated by low carbon monoxide lung diffusing capacity (DLCO), are largely undisputed to predict survival. Accordingly, the recommendations to maximize survival are of rather general nature and include cessation of smoking and daily exercises. As of yet, there are no established, specific molecular markers that reliably predict survival of COPD patients.

To help in filling this gap, in the present study we evaluated genome-wide miRNA signatures of COPD patients with regards to overall survival. Beyond their pathophysiological role, such signatures are of interest, since they bear the potential for automated, affordable, chip-based detection procedures that could become part of clinical routine. For this purpose, we compared the blood-born miRNome between COSYCONET patients who died within (*n* = 53) and patients who survived the 54-month follow-up period (*n* = 480).

## 2. Results

**Figure 1:**
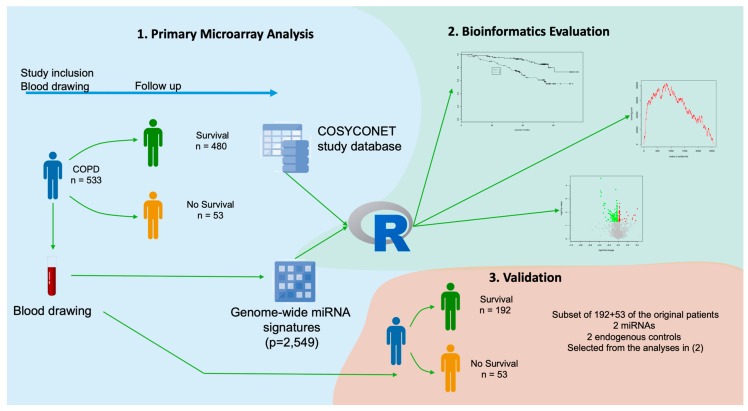
Overview of the study set up. The study consists of three parts: (**1**) a primary microarray-based screening, (**2**) a bioinformatics evaluation of the data and candidate selection, and (**3**) a validation in a subset of samples using RT-qPCR. Blood-born microRNA profiles were related to the survival of patients with chronic obstructive pulmonary disease (COPD) over a period of 54 months after diagnosis. COSYCONET: COPD and SYstemic consequences-COmorbidities NETwork.

We evaluated miRNA signatures from peripheral blood cells of COPD patients for their correlation with the patient survival. As sketched in Figure 1, the peripheral blood samples were drawn at the time of inclusion in the COSYCONET study (visit 1) and the 54 months survival was selected as the primary endpoint. In general, we followed the STARD and CONSORT criteria in performing and reporting the study.

### 2.1. miRNAs are Related to 54-Month Survival

**Figure 2:**
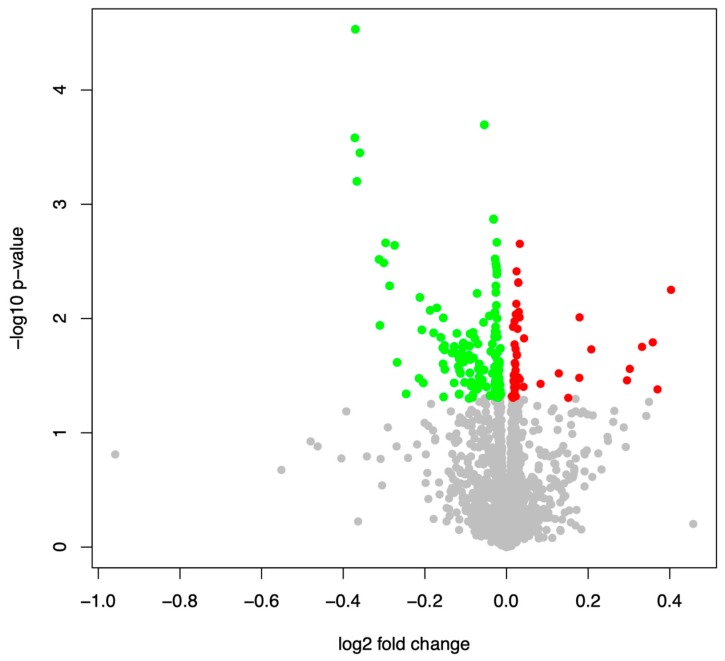
Volcano plot for the differentially expressed miRNAs in COPD patients surviving a 54-month period versus patients who died within this period. Significant miRNAs are indicated in red (overexpressed in surviving patients) or green (reduced expression in surviving patients).

We determined the blood-born miRNAs with differential abundance between patients who died within the 54 months period and the patients who survived this period, in the following referred to as “non-surviving” and “surviving” patients, respectively. Altogether, we evaluated the expression of 2,549 human miRNAs using microarray technology. Both *t*-tests and Wilcoxon Mann-Whitney tests yielded largely consistent results. In detail, 7.7% and 7.5% of all miRNAs were significantly deregulated between groups in the two tests, respectively, with nominal *p* < 0.05. The 15 miRNAs with the most significant differential abundance between surviving and non-surviving are summarized in Table S1. The complete list of all miRNAs with their *p* value and other metrics is given in Supplemental Table S1. The majority of the miRNAs with significant differences was overexpressed in the surviving patients: All but one (miR-143-5p) of the 15 miRNAs show an elevated abundance in the surviving patients; this included members of the miR-320 family (-320c, -320e, -320b, -320d) and the oncogenic miR-150-5p. The tendency towards increased miRNA abundance for the surviving patients can also be seen in Volcano plots (Figure 2). Of all 175 significant miRNAs (nominal *p* < 0.05), 126 (72%) were more strongly expressed in the surviving patients.

### 2.2. Annotation of miRNAs Correlated with Survival: Higher Expression in Immune Cells

Since the understanding of the functional role of a potential marker can substantially increase its value, we analyzed the networks in which these miRNAs exert their functions, using our miRNA enrichment tool miEAA [17]. To this end, we considered two sets of miRNAs. First, we focused on those miRNAs with elevated abundance in the surviving patients. Then, we independently investigated the miRNAs with decreased abundance in surviving patients with the same methodology. MiRNAs with reduced abundance in surviving patients were enriched for targets genes and pathways associated with various disease categories, most importantly cancers such as carcinoma acinar cell, carcinoma transitional cell, insulinoma, and lymphoma B-cell. MiRNAs with elevated abundance in surviving patients were enriched in targets genes and pathways associated with immune cells including cells expressing CD14, CD9, CD13, or CD56. The target genes of the most significant miRNAs appeared to be part of a comprehensive network, members of which have already been experimentally validated by reporter assays in different cell types [18,19,20]. The core of this network included the genes SP1 (*Sp1* transcription factor), TGFBR1 (transforming growth factor beta receptor 1), IGF1R (insulin-like growth factor 1 receptor) and GNAI1 (G protein subunit alpha i1), which are known to be targeted by members of the miR-320 family among others (Supplemental Figure S1). This accumulation of genes targeted by the miR-320 reinforces their central position as suggested already by the results presented in Table S1. The identified targets TGFBR1 and IGF1R have been shown to be downregulated in blood cells of patients with autoimmune disease [21] and a loss of SP1 and knockdown of GNAI1 have been described to impair hematopoiesis and immune cell migration capability, respectively [22,23].

### 2.3. miR-320b and miR-21-5p as Prognostic Marker for COPD by Kaplan-Meier Estimators

**Figure 3:**
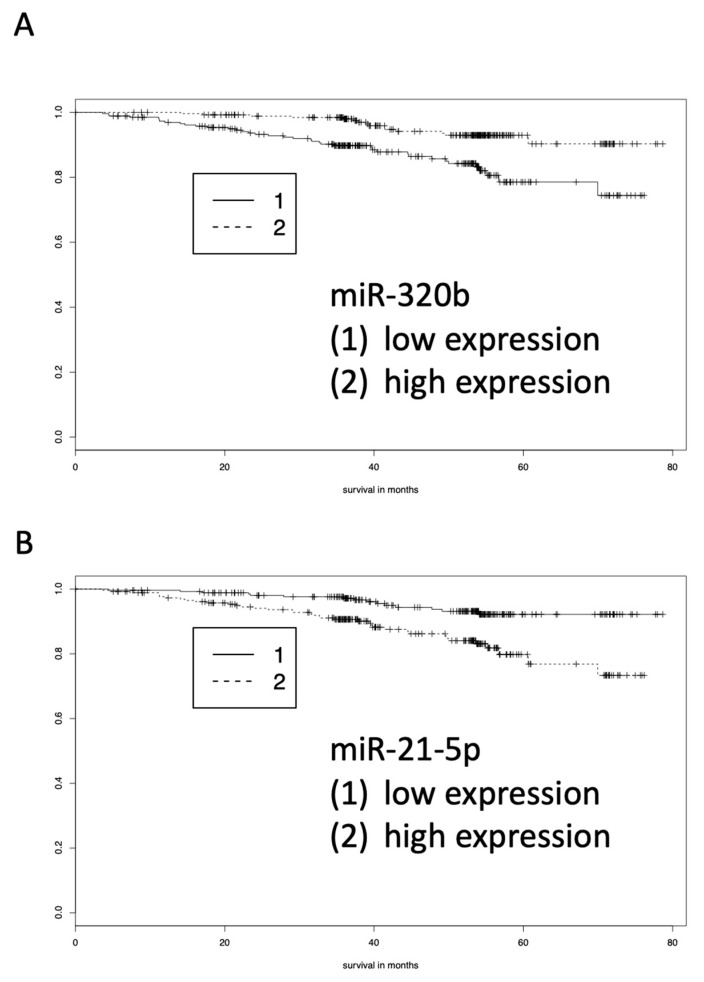
Kaplan-Meier analyses of blood-born miRNA pattern determined by array analysis. (**A**) MiR-320b indicates longer survival of COPD patients who show higher abundance of miR-320b. (**B**) MiR-21-5p indicates longer survival of COPD patients who show lower abundance of miR-21-5p.

While the above analysis focused on the abundance of blood-born miRNAs in the two patient groups, we also analyzed miRNAs for their correlation with 54 months survival. Using Kaplan-Meier estimators, six miRNAs (miR-21-5p (negative correlation with survival), miR-320b (positive correlation with survival), miR-3123 (negative correlation with survival), miR-4264 (positive correlation with survival), miR-4782-5p (positive correlation with survival), and miR-4792 (negative correlation with survival)) were identified that were significantly correlated with survival time within the 54-month period. These included miRNAs with both increased and decreased abundances, each of them associated with prolonged survival. Figure 3A,B show two examples of a miRNA with high expression (miR-320b) and a miRNA with low expression (miR-21-5p) in patients with longer survival.

### 2.4. RT-qPCR Validates miR-320b and miR-150-5p

**Figure 4:**
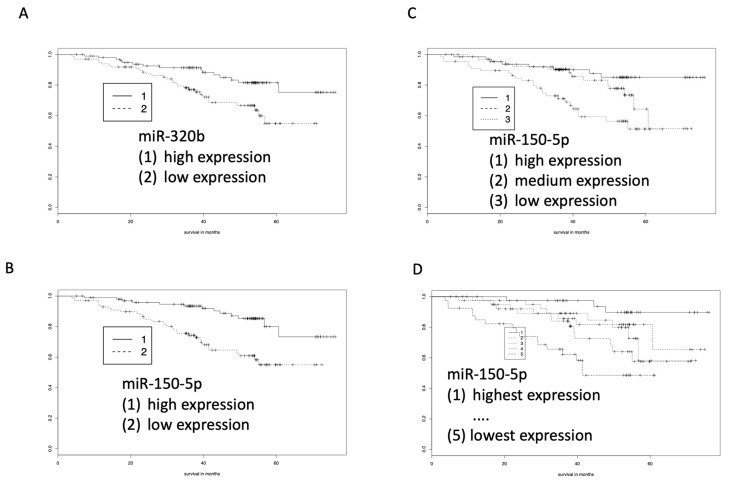
Kaplan-Meier analyses of blood-born miRNA patterns determined by RT-qPCR. (**A**) MiR-320b indicates longer survival of COPD patients who show higher abundance of miR-320b. (**B**) MiR-150-5p indicates longer survival of COPD patients who show higher abundance of miR-150-5p. (**C**) Splitting the patients in tertile groups with high, medium, and low expression of miR-150-5p showed longest survival for patients with highest expression. (**D**) Splitting in quintile groups also demonstrated the longest survival for patients with highest expression.

Using RT-qPCR, we confirmed the differences in abundance of miR-320b and miR-150-5p in 192 surviving versus 53 non-surviving patients. The two miRNAs were selected because of three criteria. First, they were among the top 15 most significant miRNAs among all tested 2549 miRNAs. Second, they showed a high abundance in our samples. Third, a PubMed search using the miRNA identifiers and “COPD” or “lung cancer” or “cancer” lead to evidence that the miRNAs have a biological function. As endogenous control, we originally used SNORD48, which has been frequently chosen in studies on miRNA expression in whole blood. We, however, also tried to identify a miRNA as endogenous control. Indeed, miR-21-3p shows a *p* value of 0.83 for the nonparametric test and a *p* value of 0.54 for the *t*-test in the array analyses, indicating that the miRNA is stably expressed at comparable levels between the groups. Thus, we also used miRNA-21-3p as endogenous control. Notably, the -5q arm of miRNA-21 was among the miRNAs that showed a highly differential abundance between surviving and non-surviving patients. Since we obtained concordant results with both controls, the subsequent analyses were done with miR-21-3p only. As shown in Figure 4A,B, the survival curves for miR-320b and miR-150-5p confirmed the results of the microarray analyses, however, the effect sizes for both miRNAs were higher in the PCR than in the microarray analyses, which was probably due to the higher sensitivity of RT-qPCR.

A partitioning of the patients in different numbers of subgroups, ranging from a group with highest expression to a group with lowest expression, led to even more clear-cut findings. For example, using three groups of miR-150-5p expression survival was longest in patients with highest expression and lowest in patients with lowest expression (Figure 4C). Likewise, splitting into five groups, i.e., quintiles, showed that the miR-150-5p expression was highly correlated with survival. While 90% of patients with the highest expression of miR-150-5p survived the 54-month follow-up, only 50% of patients with lowest expression of this miRNA survived the same period. Regarding miR-320b, about 60% of patients with low abundance survived the 54-month period, in contrast to about 80% of patients with high abundance.

## 3. Discussion

Utilizing the prospective multi-centric COSYCONET study, we correlated 533 genome-wide miRNA profiles from whole blood with the survival of COPD patients during a 54-months follow-up period. The primary analysis revealed 175 miRNAs (7.6% of 2549 miRNAs) with significant differential abundance between surviving and non-surviving patients, the majority (72%) being higher expressed in patients surviving the follow-up. Out of this large pool of marker candidates, we selected two miRNAs, miR-320b and miR-150-5p, that were differentially expressed between surviving and non-surviving patients, for confirmation by RT-qPCR. Both miRNAs were suitable for this by showing an expression markedly above the detection limits of the applied technologies. A weak expression near the detection limit often handicaps the value of markers that are statistically highly significant. Equally important was the biological role of both miRNAs. Our systems biology analysis demonstrated that both are central within a dense network of target genes, many of which have already been experimentally validated by reporter assays. Finally, both miRNAs are expressed in leucocytes. Because of the cellular composition of blood, white blood cells can be assumed to be the main source of miRNAs isolated from whole blood PAXGene samples. MiRNAs from other blood cells (e.g., erythrocytes, and circulating free miRNAs or miRNAs enclosed in vesicles) are also contained in the samples but seem to account for a minor portion of RNA. In line with this assumption, our miRNA set enrichment analysis indicated that the primary affiliation of dysregulated miRNAs was the immune system.

Cancer was among the most common causes of deaths in the cohort, most frequently lung cancer. We thus analyzed whether the two validated miRNAs were also dysregulated in lung cancer tissue. To this end, we used data generated in our previous study [24], in which we had compared miRNA expression in tumor tissue and adjacent non-cancer tissue to minimize inter-individual variation. For miR-150-5p we observed a significant downregulation (nominal p value 0.04), although only 18 tissue pairs of NSCLC (Non-Small Cell Lung Cancer) patients were available for investigation. We also found a tendency towards dysregulation for members of the miR-320 family, although the significance threshold of 0.05 was not achieved [24].

Both, miR-150-5p and miR-320b have previously been identified as anti-cancer miRNAs. These functions cannot be immediately set into relation to our findings for blood cells. It is, however, conceivable that an overexpression of miR-320b and miR-150-5p can exert an anti-proliferative effect on both blood and resident on lung cells of COPD patients. Remarkably, miR-150-5p is known to target genes related to proliferation [25,26,27,28], and miR-320b represses proliferation by targeting respective genes including c-MYC [29,30,31]. Overexpression of these miRNAs in lung tissue and blood of COPD patients may have an effect by not only suppressing tumor development in the lung but also suppressing the proliferation of immune cells. This is relevant, since the inflammatory cascade triggered by inhaled irritants, can promote tissue damage, while overexpression of miR-320b and miR-150-5p might attenuate the immune response and consequently tissue damage. As previously shown, both miRNAs are indeed overexpressed not only in blood cells but also in lung cells of COPD patients. Hence, the suppression of cancer by miR-320b and miR-150-5p may have a parallel effect on non-cancer causative factors in COPD, thereby leading to a prolonged survival in addition to the role in lung cancer which is a common cause of death in COPD patients. As indicated above, miR-150-5p and miR-320b are representatives of a large number of miRNAs that were found to be differentially expressed between survivors and non-survivors. These miRNAs comprised regulators of specific cellular pathways, such as the NF-κB pathway, the activation of which plays an important role in the development for COPD and for lung cancer in COPD patients [32].

In conclusion, our results indicate that a large number of blood-born miRNAs show differences in abundance between COPD patients surviving and not surviving a 54-month follow-up period. Of these miRNAs, miR-150-5p and miR-320, both known as anti-cancer miRNAs, were particularly relevant and central within a regulatory network including many genes, whose role has been experimentally validated in previous studies. The effects of these miRNAs on survival may be twofold. First in blood cells by a possible reduction of tissue destruction via suppression of inflammatory cascades, secondly via suppression of the development of lung cancer and other cancers associated with COPD.

## 4. Materials and Methods

### 4.1. Sample Collection and RNA Extraction

Patients were recruited within the German COPD and SYstemic consequences-COmorbidities NETwork (COSYCONET) study. All local ethics committees approved the study and all contributing patients provided written informed consent. The study was performed according to the Declaration of Helsinki. For the present analysis, we used whole blood samples of 533 patients collected in PAXgene tubes (BD Biosciences, Franklin Lakes, NJ, USA) [14] at the recruitment visit (visit 1). These 533 patients include all patients in the COSYCONET cohort that developed cancer within the follow-up period and controls (COPD patients not developing cancer) with similar age and gender distribution. Total RNA was extracted using the PAXgene blood miRNA kit (Qiagen, Hilden, Germany) according to the manufacturer’s instructions. Quantity and quality of the isolated RNA were assessed using NanoDrop-1000 (Thermo Fisher Scientific, Waltham, MA, USA) and 2100 Bioanalyzer (Agilent, Santa Clara, CA, USA). For each patient, the full miRNome (2549 miRNAs) was generated using microarray technology as described below.

### 4.2. MiRNA Expression Profiles

The miRNA expression profiles were measured using SurePrint Human miRNA microarrays V21 (Agilent, Santa Clara, CA, USA) according to the manufacturer’s recommendations and as recently published [14]. Total RNA was dephosphorylated, labeled, and hybridized to the array for 20 h at 55 °C. After washing and drying, arrays were scanned using an Agilent microarray scanner, the raw expression data was generated using the Agilent Feature Expression software. Afterwards, the raw data was quantile normalized and log-transformed using R scripts.

### 4.3. RT-qPCR Profiles

To quantify the miRNA levels of 245 blood samples from COPD patients, we used the miScript system (Qiagen, Hilden, Germany) following the manufacturer’s recommendations. In the replication, we measured samples from the original study where sufficient RNA of high quality has been available, including all patients who died during the follow-up period as well as 192 patients who survived. In short, 100 ng total RNA was reverse transcribed using the miScript RT II kit. Subsequently, quantitative real-time PCR reaction was set-up using 1 ng cDNA, the miScript SYBR Green kit and miScript Primer assays and run on a StepOnePlus PCR cycler (Thermo Fisher Scientific, Waltham, MA, USA). Expression levels of miRNAs were calculated using the **Δ Δ** Cq method [33]. We measured SNORD48 and miRNA-21-3p as endogenous controls. SNORD48 is among the most widely used standard endogenous controls. We selected miR-21-3p as our second candidate since it showed similar expression levels (not significantly deregulated) between surviving and non-surviving patients. Since the results between both endogenous controls were largely consistent the presented results rely on miR-21-3p as endogenous control.

### 4.4. Statistical Analysis

For comparing the differentially regulated miRNAs between the survival and non-survival group, we used unpaired two-tailed *t*-tests and non-parametric unpaired two-tailed Wilcoxon Mann-Whitney (WMW) tests. The non-parametric WMW test was used in addition to the *t*-test since not all miRNAs were normally distributed, one of the basic assumptions of the *t*-test. Because of the exploratory character of the study, the nominal *p* value were reported and significant miRNAs from the microarrays were further validated by RT-qPCR. Supplemental Table S1 lists however both, raw as well as adjusted (Benjamini-Hochberg) *p* value for all tested miRNAs. For the Kaplan-Meier survival analysis, we used the R package “survival” to compute the estimators for the 54-month period and to draw the survival curves. Here, all miRNAs with a nominal *p* value of below 0.001 were considered to be statistically significant. All statistical computations were done using the statistical programming language R R 3.3.2 GUI 1.68 Mavericks build (7288) (The R Foundation). 

### 4.5. miRNA Enrichment Analysis

miRNAs that showed significant up-regulation and significant down-regulation (two-tailed unpaired *t*-test, *p* < 0.05) were selected as test sets and all miRNAs measured on the Agilent array (i.e., 2549 miRNAs) as reference set for enrichment analysis with default parameters in miEAA [17].

## Supplementary Materials

The following are available online at https://www.mdpi.com/2073-4409/8/10/1162/s1, Figure S1: Network of miRNAs with elevated abundance in surviving patients. mRNA-targets that are predicted for two miRNAs are indicated in blue and mRNAs that are predicted to be targeted by three miRNAs are indicated in orange. SP1: *Sp1* transcription factor, TGFBR1: transforming growth factor beta receptor 1, IGF1R: insulin-like growth factor 1 receptor, GNAI1: G protein subunit alpha i1., Table S1: The 15 miRNAs with the most significant differential abundance between the surviving and the non-surviving patient groups.
