# Supplementary material for: Low miR-150-5p and miR-320b Expression Predicts Reduced Survival of COPD Patients
Source: Cells. 2019 Sep 27;8(10):1162. doi: 10.3390/cells8101162 (PMC6848926; doi:10.3390/cells8101162)
Supplement: Supplementary file 1 [file cells-08-01162-s001.pdf]

Supplemental Materials

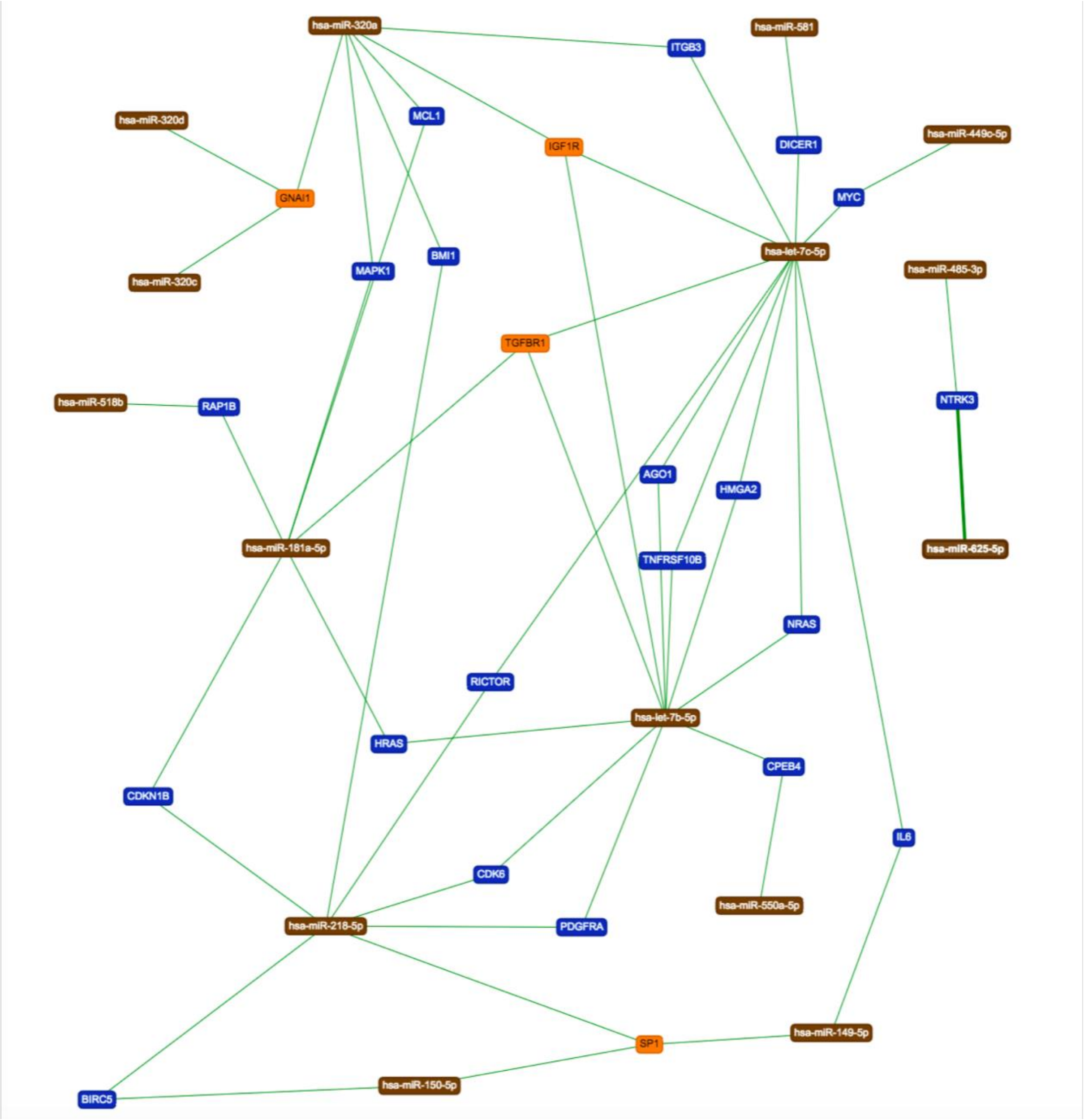

Figure S1: Network of miRNAs with elevated abundance in surviving patients. mRNA-targets that are predicted for two miRNAs are indicated in blue and mRNAs that are predicted to be targeted by three miRNAs are indicated in orange. *SP1*: Sp1 transcription factor), *TGFBR1*: transforming growth factor beta receptor 1, *IGF1R*: insulin like growth factor 1 receptor, *GNAI1*: G protein subunit alpha i1.

Table S1: The 15 miRNAs with the most significant differential abundance between the surviving and the non-surviving patient groups.

miRNA	median expression non-surviving	median expression surviving	WMW test p-value	t-Test p-value
miR-320c	901.66	1108.97	0.00295	0.00003
miR-6827-3p	11.06	11.5	0.00133	0.00020
miR-320e	1443.27	1889.94	0.00740	0.00026
miR-320b	1958.99	2402.51	0.00322	0.00036
miR-320d	2039.41	2509.03	0.01582	0.00063
miR-5011-3p	9.79	9.97	0.00162	0.00134
miR-203b-5p	10.03	10.26	0.00138	0.00136
miR-4330	9.4	9.48	0.01270	0.00215
miR-4732-5p	70.3	84.75	0.02095	0.00219
miR-143-5p	10.37	10.14	0.00133	0.00223
miR-218-5p	10.52	10.55	0.29121	0.00230
miR-6874-3p	9.7	9.85	0.00468	0.00301
miR-150-5p	9357.82	12063.35	0.00148	0.00304
miR-181a-5p	426.35	486.41	0.05056	0.00326
miR-3126-3p	9.49	9.7	0.00168	0.00332
